# A Novel Allele Specific Polymerase Chain Reaction (AS-PCR) Assay to Detect the V1016G Knockdown Resistance Mutation Confirms Its Widespread Presence in *Aedes albopictus* Populations from Italy

**DOI:** 10.3390/insects12010079

**Published:** 2021-01-17

**Authors:** Verena Pichler, Emiliano Mancini, Martina Micocci, Maria Calzetta, Daniele Arnoldi, Annapaola Rizzoli, Valeria Lencioni, Francesca Paoli, Romeo Bellini, Rodolfo Veronesi, Simone Martini, Andrea Drago, Claudio De Liberato, Arianna Ermenegildi, Joao Pinto, Alessandra della Torre, Beniamino Caputo

**Affiliations:** 1 Dipartimento di Sanità Pubblica e Malattie Infettive, Università Sapienza, 00185 Rome, Italy; verena.pichler@uniroma1.it (V.P.); micocci.1449940@studenti.uniroma1.it (M.M.); maria.calzetta@uniroma1.it (M.C.); 2 Dipartimento di Biologia e Biotecnologie ‘C. Darwin’, Università Sapienza, 00185 Rome, Italy; emiliano.mancini@uniroma1.it; 3 Research and Innovation Centre, Department of Biodiversity and Molecular Ecology, Fondazione Edmund Mach, San Michele all’Adige, 38098 Trento, Italy; daniele.arnoldi@fmach.it (D.A.); annapaola.rizzoli@fmach.it (A.R.); 4 Section of Invertebrate Zoology and Hydrobiology, MUSE-Science Museum, 38098 Trento, Italy; Valeria.Lencioni@muse.it (V.L.); Francesca.Paoli@muse.it (F.P.); 5 Centro Agricoltura Ambiente “G. Nicoli”, 40014 Crevalcore, Italy; rbellini@caa.it (R.B.); rveronesi@caa.it (R.V.); 6 Entostudio snc, 35020 Padua, Italy; martini@entostudio.com (S.M.); drago@entostudio.com (A.D.); 7 Istituto Zooprofilattico Sperimentale del Lazio e della Toscana “M. Aleandri”, 00178 Rome, Italy; claudio.deliberato@izslt.it (C.D.L.); arianna.ermenegildi@izslt.it (A.E.); 8 Global Health and Tropical Medicine, Instituto de Higiene e Medicina Tropical, Universidade Nova de Lisboa, 1349-008 Lisboa, Portugal; JPinto@ihmt.unl.pt

**Keywords:** *Aedes albopictus*, insecticide resistance, integrated vector management, vector control, *kdr* genotyping, pyrethroid resistance

## Abstract

**Simple Summary:**

The Asian Tiger mosquito, *Aedes albopictus*, is an invasive species which has become a worldwide public health concern due to its colonization of all continents (except Antarctica), its aggressive biting behavior and its capacity to transmit potentially deadly human viruses, such as Dengue and Chikungunya. Insecticides currently represent the most commonly used weapon to control epidemics of mosquito-borne viruses, but their effectiveness is threatened by the fast and worldwide spread of resistant mosquito vector populations. Molecular approaches able to easily detect mosquito genetic traits associated with insecticide resistance are among the key tools to counteract this phenomenon. We developed and tested a method that makes it possible to detect the presence in *Aedes albopictus* of a specific genetic trait (the so-called knock-down resistance (*kdr*) mutation) associated with resistance to pyrethroids, the most commonly used insecticidal class. We tested this approach on mosquitoes sampled across Italy and show that the *kdr* mutation is widespread in the country and reaches worrying frequencies (up to 45%) in coastal areas where pyrethroids are widely exploited to reduce mosquito nuisance. These results should serve as a warning bell and encourage further studies to inform insecticide management policies with the aim of maintaining the effectiveness of pyrethroids in the long term.

**Abstract:**

Polymerase chain reaction (PCR)-based genotyping of mutations in the voltage-sensitive sodium channel (*vssc*) associated with resistance to pyrethroid insecticides is widely used and represents a potential early warning and monitoring system for insecticide resistance arising in mosquito populations, which are vectors of different human pathogens. In the secondary vector *Aedes albopictus*—an Asian species that has invaded and colonized the whole world, including temperate regions—sequencing of domain II of the *vssc* gene is still needed to detect the V1016G mutation associated with pyrethroid resistance. In this study we developed and tested a novel allele-specific PCR (AS-PCR) assay to genotype the V1016G mutation in this species and applied it to the analysis of wild populations from Italy. The results confirm the high accuracy of the novel AS-PCR and highlight frequencies of the V1016G allele as >5% in most sampling sites, with peaks of 20–45% in coastal touristic sites where pyrethroid treatments are extensively implemented, mostly for mosquito nuisance reduction. The high frequency of this mutation observed in Italian *Ae. albopictus* populations should serve as a warning bell, advocating for increased monitoring and management of a phenomenon which risks neutralizing the only weapon today available to counteract (risks of) arbovirus outbreaks.

## 1. Introduction

Vector-borne diseases account for approximately 17% of the estimated global burden of all infectious diseases and cause more than 700,000 deaths each year [1]. Mosquito-borne viruses such as Dengue have undergone an extraordinary global spread, with a 30-fold increase in incidence during the last 50 years [1,2], causing almost 100 million cases/year in >100 tropical countries. Moreover, several arboviruses have also experienced a (re-)emergence in temperate regions, mostly due to increased international travel and trade which have favored the movement of infected travelers along with disease vectors [3], the latter finding further favorable conditions for establishment and colonization thanks to global warming [4,5]. In particular, the exceptional expansion of the range of the highly invasive mosquito vector *Aedes albopictus* during the last 30 years has caused a Dengue outbreak with more than 37,000 human cases in China in 2014 [6], several autochthonous Dengue and Chikungunya cases in Europe in the last decade [7,8,9,10,11], and two Chikungunya outbreaks in Italy in 2007 and 2017 with more than 200 and 500 human cases, respectively [12,13,14].

Pyrethroids, a class of synthetic insecticides characterized by a high level of effectiveness against target species and low acute toxicity to vertebrates, remain the first choice for chemical-based vector control [15]. Due to their longstanding and widespread use, resistance to pyrethroids (PyR) in mosquito vector populations has become a great concern, particularly with regard to species producing the highest public health impact, such as main Afrotropical malaria vectors and the most efficient tropical arbovirus vector, *Aedes aegypti* [16,17,18]. In temperate countries, although pyrethroids are recommended only to control/interrupt outbreaks of exotic arboviruses, they are also widely employed to reduce mosquito density and nuisance [19,20,21].

The insecticidal activity of pyrethroids is based on the slowing down of the voltage-sensitive sodium channel (VSSC) gating kinetics and of the nervous signal transmission, resulting in a fast knock-down effect on mosquitoes, which become unable to fly or move in a coordinated way [22,23,24]. One of the major PyR mechanisms results from mutations at target sites in the VSSC, causing reduced interaction with the insecticides. Several of these mutations in the *vssc* gene causing reduced susceptibility to pyrethroids—known as knock-down resistance (*kdr*) mutations—are currently widespread in mosquito vectors, due to the strong selective pressure exerted by pyrethroid use against mosquito populations, as well as against other insect pests [16,17,25]. Polymerase chain reaction (PCR)-based approaches make it possible to easily detect *kdr* mutations in wild populations of malaria vectors [26] and of *Ae. aegypti* [27,28]. Such molecular assays are becoming fundamental tools for monitoring the occurrence and spread of target site mutations associated with insecticide resistance. They can be easily applied to large samples composed of different developmental stages, sexes, or ages, without requiring breeding of live mosquitoes, as in the case of susceptibility bioassays.

Increasing levels of PyR are reported also in *Ae. albopictus*, both in its native and invasive range, with particular reference to the Indian subcontinent and Africa [29,30,31,32,33,34,35,36,37,38,39]. There is also growing evidence for PyR emerging in *Ae. albopictus* invasive populations in temperate regions, such as Italy, Greece, Spain, and the USA [40,41,42,43,44,45].

So far, *kdr* mutations identified in this species involve three amino acidic positions of the VSSC, i.e., e I1532T [18,45,46], F1534C/L/S [33,46,47,48], and V1016G [42,45,49]. The latter was shown to confer stronger PyR, compared to mutations in position 1534, when exposing homozygous lab strains to different classes of pyrethroids [49]. While the widespread presence of mutations in position 1534 has been assessed [18,45,46,50] and can be monitored thanks to the locus-specific genotyping PCR-approach developed by Zhu et al. [51], monitoring V1016G still requires a more demanding sequencing approach of VSSC domain II. This has so far made it possible to highlight the widespread presence of the V1016G mutation in *Ae. albopictus* populations from Italy and its presence in populations from La Reunion (France), Hanoi (Vietnam), and Guangzhou (China) [42,49,50,52].

We here propose a straightforward and reliable allele-specific PCR (AS-PCR) -assay to detect the V1016G mutation in *Ae. albopictus* that will significantly contribute to assessing PyR in this species across its native and invasive range. We applied the novel AS-PCR to analyze wild populations from north and central Italy and revealed frequencies up to 45% in coastal touristic sites where pyrethroid treatments have been implemented for several years to reduce mosquito nuisance.

## 2. Materials and Methods

### 2.1. Design of Aedes Albopictus V1016G AS-PCR Assay

**Figure 1:**
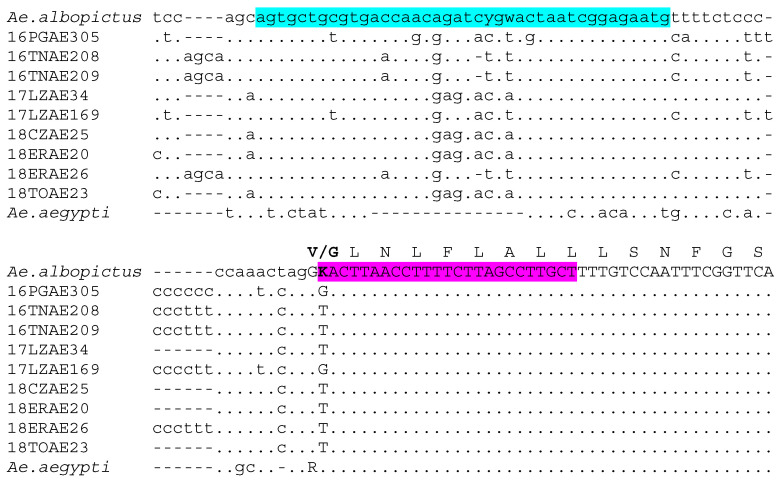
Partial alignment of domain II of the voltage-sensitive sodium channel genes of *Ae. aegypti* and *Ae. albopictus*. Intronic sequences are shown in lower case and mutation V1016G in bold in both the nucleotidic sequence and the above amino acidic sequence. Purple: primer binding site of allele-specific reverse primers. Light blue: position of the newly designed primer Albo1016for.

The allele-specific PCR assay for rapid identification of *Ae. albopictus* specimens carrying the wildtype (Valine; 1016V) or mutated (Glycine; 1016G) kdr allele in position 1016 of the *vssc* gene was designed as follows. A 40-bp long species-specific forward primer (Albo1016for 5′-AGTGCTGCGTGACCAACAGATCYGWACTAATCGGAGAATG-3′) was designed based on the alignment (obtained using a muscle algorithm with standard parameters in MEGA X [53]) of a partial fragment of exon 21 of the *vssc* gene of *Ae. albopictus* specimens sequenced in the frame of the work described in Pichler et al. [40], as well as a sequence obtained from vectorbase [54] (Supercontig:AaloF1:JXUM01S000562:547479-548121) (Figure 1). Given the high sequence homology of the region of exon 21 surrounding the V1016G kdr mutation between *Ae. albopictus* and *Ae. aegypti* (AaegL5_3:315983683-315983977; [54]) (Figure 1), we used a pair of V1016G allele-specific reverse primers formerly designed for a RT-PCR kdr mutation genotyping approach in *Ae. aegypti* [28] and then applied in an AS-PCR protocol [27] on the same species. Both reverse primers (Gly1016rev: 5′-GCGGGCAGGGCGGCGGGGGCGGGGCCAGCAAGGCTAAGAAAAGGTTAAcTC-3′; Val1016rev: 5′-GCGGGCAGCAAGGCTAAGAAAAGGTTAAtTA-3′) contain intentional mismatches at 3′ end (in lowercase), enhancing specificity [55] at targeted mutations (in bold), and GC-rich tails of varying lengths (underlined) to generate amplified products distinguishable by a difference of 20 bp in size.

To avoid carry-over PCR contamination that frequently led to amplification of undesired faint bands (as also in negative controls) during the preliminary testing on 23 specimens previously genotyped by sequencing [42], we employed the Uracil-N-glycosylase (UNG)-dUTP approach [56]. This method consists of incorporating dUTP in all newly synthesized PCR products (by substituting dTTP with dUTP) to render them susceptible to the hydrolysis by the bacterial enzyme UNG. The addition of this enzyme to the subsequent reaction mixture allows the selective hydrolysis and removal of the possible contaminating amplicons (including misprimed and nonspecific products) from the PCR mix, with no effect on natural (i.e., thymine-containing) genomic DNA, which serves as a unique template.

Each reaction was performed in a 25 μL volume consisting of: 1× PCR Reaction Buffer (Bioline, MEM, TN, USA), 4.0 mM MgCl_2_, 1 unit BioTaq polymerase (Bioline, MEM, TN, USA), 0.2 mM each of dATP, dCTP, dGTP, and 0.4 mM of dUTP (ThermoFisher Scientific, WLM, MA, USA), 0.25 μL of Cod Uracil-DNA Glycosylase (Cod UNG; Arcticzymes; Tromsø, Norway), 0.4 μM of primers Albo1016for and Gly1016rev and 0.25 μM of primer Val1016rev, and 1.5 μL of template DNA (~1–2 ng).

**Figure 2:**
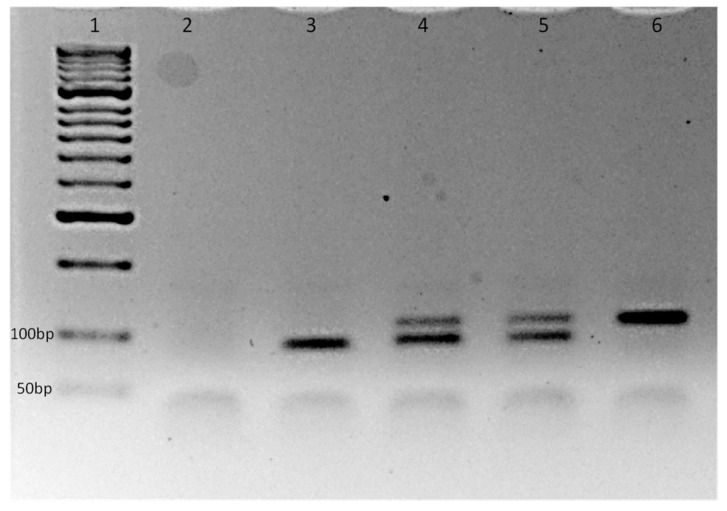
Representative electrophoretic profiles of the allele-specific polymerase chain reaction (AS-PCR) for the detection of the knock-down resistance (*kdr*) allele 1016G in in the *vssc* gene in *Aedes albopictus* specimens. Agarose gel 3%. Lane 1: molecular weight marker (50 bp, Bioline, Memphis, TN, USA); lane 2: negative control; lane 3: homozygote 1016V/1016V; lanes 4 and 5: heterozygote 1016V/1016G; lane 6: Homozygote 1016G/1016G.

Thermocycler conditions included a pre-incubation step for optimal UNG activity at 37 °C for 5 min, followed by an initial DNA denaturation step at 94 °C for 2 min. Afterward, 35 cycles at 95 °C for 30 s, 54 °C for 30 s and 72 °C for 20 s were employed with a final elongation at 72 °C for 3 min. PCR amplification products were loaded onto a 3% agarose gel stained with 6.5 μL Midori Green Advance (Nippon Genetics, Tokyo, Japan) and run for 30–40 min at 85 V in 1xTBE buffer. The assay produced a ~90 bp fragment for the wildtype (susceptible: 1016V) allele and a 110 bp fragment for the mutant (resistant: 1016G) one (Figure 2).

### 2.2. Validation of the Novel AS-PCR Assay on Field Samples from Italy

The PCR assay was applied to genotype *Ae. albopictus* specimens collected in 36 sites from the Trentino, Veneto, Emilia Romagna, Toscana, and Lazio regions in Italy (Table 1); in all these regions, with the exception of Trentino, the presence of the V1016G mutation was already reported [42,49,50]. Sampling was performed between May and October in 2019 and 2020 with either BG-Sentinel traps or ovitraps. In the latter case at least five traps per sampling site were employed to avoid oversampling of siblings and eggs were then mailed to the Department of Public Health and Infectious Diseases at Sapienza University in Rome, where they were hatched and reared to adults under insectary conditions (T = 26 ± 1 °C; RH = 60 ± 5%; 14:10 h light:dark photoperiod). DNA was extracted from legs of single mosquitoes (485 individuals) using the DNAzol^®^ DNA extraction reagent (TermoFisher Scientific, USA) following Rider et al. [57] and eluted in 30 μL ddH_2_O. For a subset of 39 individually genotyped specimens, AS-PCR results were compared with partial sequences of domain II of the *vssc* gene obtained following the PCR protocol described by Kasai et al. [47] with successive modifications [49]. PCR products were purified using the SureClean Kit (Bioline, USA) and sequenced at BMR Genomics s.r.l. (Padua, Italy, Genbank accession numbers: MW375084-MW375122).

The accuracy of the AS-PCR was estimated as the number of correct assessments divided by the total number of observations, taking the DNA sequencing results as the golden standard. Chi-squared or Fisher’s exact tests were performed to compare the results obtained for neighboring sampling sites characterized by either high or low/no reported pyrethroid treatments, where this information was available (Table 1), and goodness-of-fit tests for Hardy–Weinberg (HW) expectations were performed considering samples from the same region as one population.

To facilitate its exploitation in large scale studies, V1016G AS-PCR was also tested on pools of multiple specimens with a known genotype, including as a DNA template 5 μL of DNA from two, three, or four homozygote-susceptible (1016V/1016V) specimens and from one heterozygote (1016G/1016V) specimen. The PCR on each pool was replicated at least three times to assure repeatability.

## 3. Results

### 3.1. The Novel Aedes Albopictus V1016G AS-PCR Assay

The novel AS-PCR assay unambiguously genotyped 98.7% of the 485 individually analyzed field-collected Ae. albopictus specimens. Validation of the resulting AS-PCR genotypes by sequencing of a ~500 bp fragment of domain II of the vssc gene, including the V1016G locus, on 39 specimens led to an estimated accuracy of 95% (Table 2). Out of the two incorrectly genotyped specimens, one was genotyped as 1016V/1016V homozygote by sequencing, but as heterozygote by the PCR assay, probably due to an aspecific binding of the allele-specific primer Gly1016rev, since no other polymorphisms were observed in primer binding sites. The second incorrectly genotyped specimen was genotyped as heterozygote by sequencing, but as homozygote 1016G/1016G by the AS-PCR assay: this specimen carried the two last positions of the primer binding site in heterozygosis; the resulting codons did not affect the amino acidic sequence, resulting in a 1016V/1016G heterozygote, but probably caused a suboptimal binding of the primer Val1016rev. Silent mutations in the triplet coding position 1016 of the vssc gene were detected in three further specimens, all of which resulted in homozygotes 1016V/1016V, but with codon GTG instead of GTA for the susceptible allele. In none of these cases did the sequence polymorphism affect the AS-PCR assay results.

Testing the novel AS-PCR on 18 pools made it possible to obtain clear and replicable banding patterns when pools included one heterozygote (1016G/1016V) and two homozygote-susceptible (1016V/1016V) ones. Results of the AS-PCR (as well as of sequencing) were instead ambiguous and less consistent among replicates when one heterozygote was diluted within more than two homozygote specimens.

### 3.2. Frequency of 1016G kdr Allele in Aedes Albopictus Field Populations from Italy

**Figure 3:**
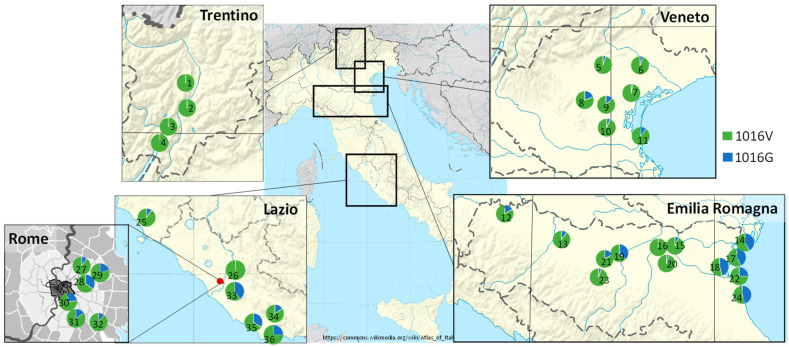
Allele frequency for wildtype (1016V = green) and mutated (1016G = blue) alleles in position 1016 of the *vssc* gene in *Aedes albopictus* populations from 36 sites across five Italian regions. Numbers identify sample sites according to Table 1. Map modified from map created by Wikimedia Commons users Sting and NordNordWest; CC BY-SA 3.0.

Overall, 479 field collected specimens from the Trentino (60 specimens from four sites), Veneto (119 from seven sites), Emilia Romagna (170 specimens from 13 sites), Lazio (114 from 11 sites), and Toscana (16 from one site) regions were successfully genotyped by the novel AS-PCR (Table 1 and Figure 3). The 1016G allele was present in all sampled sites, except in Trentino (all four sites), one site in Emilia Romagna (site 16) and one in Lazio region (site 26). Although small sample sizes require caution with regard to the significance of the frequencies obtained, it is noteworthy that in Emilia Romagna the allele associated with reduced susceptibility (1016G) reached frequencies above 40% in most coastal sites, which are reported to be heavily treated with pyrethroids to reduce mosquito nuisance, and <20% in most of the less heavily treated inland sites (chi-square for allelic frequency in sites 14, 17, 18, 22, and 24 vs. all the others: 36.12; df = 1; *p* = <.0001; Fisher’s exact test for genotypic frequencies: *p* < 0.0001). A similar, although not significant, trend can be observed in Veneto where frequencies of the 1016G allele were >10% only in sites with reported pyrethroid treatments (chi square for allelic frequency: 1.6; df = 1; *p* = >0.2; Fisher’s exact test for genotypic frequencies: *p* > 0.15). In Lazio and Toscana regions, frequencies of the 1016G allele ranged from 0 to 40%, but a lack of reliable information on pyrethroid usage did not allow speculation as to a possible selective role of adulticide treatments. No significant deviation from the Hardy–Weinberg equilibrium was detected in any of the examined regions.

## 4. Discussion

### 4.1. Aedes Albopictus V1016G AS-PCR Assay

We here present the first AS-PCR assay to easily detect the V1016G *kdr* mutation in the VSSC protein associated with resistance to pyrethroids in *Aedes albopictus* [49]. The novel method shows a high accuracy and has the potential to become a widespread and affordable tool for early warning of resistance to pyrethroid insecticides in this highly invasive species. High accuracy of AS-PCR results when applied to pools of three specimens (with only one of which carrying the V1016G mutation at the heterozygous state) will further facilitate the application of this assay in large-scale studies.

The two discrepancies observed between the AS-PCR and DNA sequencing results can be explained by intrinsic possible problems of AS-PCR assays; i.e., (1) the possible amplification of both matched and mismatched alleles in some suboptimal situations [58], and/or (2) the failure of amplification of one of the two alleles due to polymorphisms in primer binding sites. Indeed, sequencing confirmed the presence of at least one silent mutation coding position 1016 of the VSSC protein, with Valine being coded by codon GTG instead of GTA. Remarkably, in the analyzed sample both erroneous identifications did not preclude the detection of the 1016G *kdr* allele, but slightly overestimated its presence, a problem that can be overcome by confirming the resistant allele by sequencing.

Notably, however, the designed AS-PCR faced a couple of other technical issues which need to be highlighted. Firstly, the target codon lies closely to an intron showing large variability in nucleotide diversity and length (Figure 1). This could affect the annealing of the universal forward primer (Albo1016for), as well as the length of the amplified product. In order to prevent/limit interferences with the primer annealing, we ensured efficient binding and primer elongation by avoiding polymorphisms at the 5′ and 3′ of the primer, and included two degenerate nucleotides in Albo1016for. In addition, the 40 bp-length of Albo1016for guarantees a strong binding even in the presence of few mismatches. In fact, the results showed the ability of the assay to detect the 1016G *kdr*-allele even in the latter case. On the other hand, the variability in length of the intron (in particular due to insertion of stretches of T and C (as shown in Figure 1) implicates small (i.e., ~10 bp) but visible differences in the amplicon length. These, however, did not interfere with the possibility of discriminating between the 1016V and 1016G alleles, which are identified based on amplicons ranging from 87 to 97 bp and from 107–117 bp, respectively.

Secondly, despite allele-specific reverse primers being situated within the coding region of *vssc* gene where genetic variability is expected to be low, at least one silent mutation was observed in the triplet coding for the position 1016 of *vssc* gene. The presence of additional mutations within the primer binding sites which could cause potential null alleles cannot be ruled out based on the present data.

The above potential technical artifacts impose a need for caution when applying the novel AS-PCR to the analysis of samples from unexplored geographical region, where intron polymorphisms could exceed those observed in Italy. In these situations, it is strongly recommended to confirm a subset of the AS-PCR results by DNA sequencing, particularly when recording the presence of the mutated allele for the first time. However, it is noteworthy that, since Italian populations have been shown to be a mixture of several different source populations from various parts of the world [59,60,61], the relatively limited genetic variability observed at this locus may be reassuring as to the reliability of the here-proposed genotyping assay with regard to other *Ae. albopictus* populations worldwide.

### 4.2. Presence of 1016G kdr Allele in Aedes Albopictus Field Populations from Italy

The present results (Table 1, Figure 1) confirm the widespread presence of the V1016G mutation in *Ae. albopictus* populations from Italy (already reported by Kasai et al. [42,49,50], with the exception of the extreme north-east region of Trentino. In the bordering southern Veneto region, the *kdr* allele 1016G showed frequencies ranging from 2 to 20%, with highest values in the Padova urban site. The highest frequencies (up to 45.8%) of the 1016G allele were found in coastal sites of the neighboring Emilia Romagna. In the Lazio and Toscana regions in central Italy, 1016G frequencies ranged from 0% (in a single site) to 40%.

While these results need to be interpreted with caution due to the limited sample size analyzed, observed differences in *kdr* allele frequencies may reflect differences in insecticide treatment policies at the regional and/or local level. Lack of evidence of the allele in Trentino is in fact consistent with pyrethroid spraying for mosquito control being strongly discouraged in this region, where adulticide treatments may be made only sporadically by private citizens or owners of touristic structures to reduce mosquito nuisance. The significantly higher frequencies observed instead in coastal sites, in particular in the Emilia Romagna region, are consistent with the reported heavy pyrethroid treatments ongoing for many years, mainly used to reduce the negative impact of *Aedes caspius* on tourism [21,62]. Notably, individuals carrying the 1016G allele in homozygosis were found only in the latter coastal sites. It is also worth noting here that frequencies of the V1016G mutation >20% were found in areas of coastal Emilia Romagna and Lazio affected by the 2007 and 2017 Chikungunya outbreaks respectively, supporting a possible causality between the intensive use of pyrethroid treatments to control the outbreaks and the increased pyrethroid resistance in the sites, as already hypothesized by Pichler et al. [42] for the Lazio region.

## 5. Conclusions

This study proposed a novel and reliable AS-PCR method to unambiguously genotype V1016G *kdr* mutation associated with PyR in *Ae. albopictus*, an arbovirus vector species whose worldwide distribution is expanding the risk of exotic arbovirus autochthonous transmission outside tropical areas. The availability of a novel AS-PCR to monitor PyR in field *Ae. albopictus* populations will provide the possibility of easily detecting the insurgence of the mutation at its early stage and monitoring its spread. Together with PCR-based methods to detect other *kdr* mutations (such as those available for *An. gambiae* and *Ae. aegypti* [26,27,63]), the novel AS-PCR offers the possibility of monitoring PyR in *Ae. albopictus* in order to feed insecticide resistance management policies at the international, national, and local levels, as already under way in the case for major mosquito vector species. It should however be highlighted that *kdr* mutations are only one among several mechanisms of PyR in mosquitoes and that biochemical and molecular assays, as well as more challenging bioassays, are required to obtain a complete picture of the phenotypic resistance to insecticides of a given population.

## Figures and Tables

**Table 1:** Genotype and allele frequency for wildtype (1016V = V) and mutated (1016G = G) alleles in position 1016 of the *vssc* gene in *Aedes albopictus* field populations across Italy (listed from north to south). Pyrethroid treatments were defined as high when pyrethroid spraying was performed as a routine fortnightly or monthly throughout the whole mosquito season or as low when treatments where performed occasionally resulting in two treatments maximum throughout the mosquito season.

		Site	Coordinates		Sampling Year	Treatment	N	Genotype Frequency			Freq.
			Lat	Long				GG	VG	VV	1016G
**Trentino**	1	Mezzolombardo	46°12′44.03″ N	11°5′45.77″ E	2020	NA	10	-	-	1.000	0.000
	2	Trento	46°4′8.56″ N	11°7′16.98″ E	2020	NA	30	-	-	1.000	0.000
	3	Arco	45°55′2.49″ N	10°54′36.52″ E	2020	NA	10	-	-	1.000	0.000
	4	Riva del Garda	45°51′51.46″ N	10°50′37.80″ E	2020	NA	10	-	-	1.000	0.000
		**Total**					**60**	**-**	**-**	**1.000**	**0.000**
**Veneto**	5	Castel Franco	45°40′56.19″ N	11°55′23.99″ E	2019	NA	20	-	0.100	0.900	0.050
	6	Treviso	45°39′41.48″ N	12°15′32.61″ E	2019	Low	18	-	0.111	0.889	0.056
	7	Mestre	45°28′12.36″ N	12°13′28.64″ E	2019	NA	20	-	0.050	0.950	0.025
	8	Padova	45°23′58.41″ N	11°50′20.42″ E	2019	High	13	-	0.385	0.615	0.192
	9	Legnaro	45°21′16.09″ N	11°57′4.04″ E	2019	Low	12	-	0.250	0.750	0.125
	10	Brugine	45°17′5.82″ N	11°59′52.53″ E	2019	Low	16	-	0.125	0.875	0.063
	11	Chioggia	45°12′13.93″ N	12°17′16.21″ E	2019	High	20	-	0.200	0.800	0.100
		**Total**					**119**	**-**	**0.160**	**0.840**	**0.080**
**Emilia Romagna**	12	Piacenza	45°03′08″ N	9°41′36″ E	2019	Low	12	-	0.333	0.667	0.167
	13	Parma	44°47′57″ N	10°19′34″ E	2019	low	21	-	0.190	0.810	0.095
	14	Lido di Volano	44°47′44″ N	12°15′46″ E	2019	Yes	13	-	0.846	0.154	0.423
	15	Malalbergo	44°43′09.17″ N	11°31′53.54″ E	2019	High	15	-	0.133	0.867	0.067
	16	Ponticelli	44°41′55.15″ N	11°28′24.20″ E	2019	Low	16	-	-	1.000	0.000
	17	Comacchio	44°41′41″ N	12°10′54″ E	2019	High	12	-	0.833	0.167	0.417
	18	Porto Garibaldi	44°40′40″ N	12°14′40″ E	2019	High	12	-	0.917	0.083	0.458
	19	Modena Nord	44°40′0.08″ N	10°54′47.42″ E	2019	Low	12	0.083	0.583	0.333	0.375
	20	Altedo	44°39′48.35″ N	11°30′11.02″ E	2019	Low	12	-	0.083	0.917	0.042
	21	Modena Nord-Ovest	44°39′25.72″ N	10°56′54.87″ E	2019	Low	12	-	0.333	0.667	0.167
	22	Lido di Spina	44°39′05″ N	12°14′56″ E	2019	High	12	0.083	0.333	0.583	0.250
	23	Maranello	44°31′51″ N	10°52′07″ E	2019	Low	10	-	0.100	0.900	0.050
	24	Marina Romea	44°29′00″ N	12°16′00″ E	2019	High	11	0.182	0.545	0.273	0.455
	**Total**						**170**	**0.024**	**0.382**	**0.594**	**0.215**
**Toscana**	25	Grosseto	42°45′23.8″ N	11°05′53.7″ E	2020	NA	16	-	0.187	0.813	0.094
	**Total**						**16**	**-**	**0.187**	**0.813**	**0.094**
**Lazio**	26	Guidonia	41°56′04.75″ N	12°40′00.02″ E	2020	NA	10	-	-	1.000	0.000
	27	Roma Pertini	41°55′17.14″ N	12°32′30.11″ E	2020	NA	10	-	0.200	0.800	0.100
	28	Roma-Villa Mirafiori	41°55′08.1″ N	12°31′02″ E	2020	NA	10	0.100	0.500	0.400	0.350
	29	Roma Pietralata	41°55′02.22″ N	12°33′18.83″ E	2020	NA	10	-	0.400	0.600	0.200
	30	Roma CTO	41°51′29.82″ N	12°29′13.85″ E	2020	NA	11	-	0.455	0.545	0.227
	31	Roma Appio Latino	41°51′14.51″ N	12°30′15.85″ E	2020	NA	10	0.100	0.100	0.800	0.150
	32	Roma-Appia Pignatelli	41°50′48.20″ N	12°32′55.40″ E	2020	NA	10	-	0.200	0.800	0.100
	33	Ciampino	41°47′56,24″ N	12°36′16,54″ E	2020	NA	10	0.300	0.200	0.500	0.400
	34	Fondi	41°21′26.51″ N	13°25′37.70″ E	2020	NA	10	-	0.300	0.700	0.150
	35	Sabaudia	41°18′07.56″ N	13°01′25.59″ E	2020	NA	9	0.111	0.444	0.444	0.333
	36	Terracina	41°17′05.45″ N	13°14′44.33″ E	2020	NA	14	-	0.500	0.500	0.250
	**Total**						**114**	**0.053**	**0.307**	**0.640**	**0.206**
	**TOTAL**						**479**	**0.021**	**0.255**	**0.724**	**0.148**

**Table 2:** Comparison of genotyping results obtained by sequencing the V1016G kdr mutation of the *vssc* gene in *Aedes albopictus* (rows) and with the novel AS-PCR assay (columns). Discordances are shown in italics. V = 1016V wildtype allele; G = 1016G *kdr* allele.

		AS-PCR			
		GG	VG	VV	TOT
**Sequencing**	**GG**	5			5
	**VG**	*1*	13		14
	**VV**		*1*	19	20
	**TOT**	6	14	19	39

## Data Availability

Sequence data are available in a publicly accessible repository: Genbank accession numbers: MW375084-MW375122.
